# CASINO: Surgical or Nonsurgical Treatment for cervical radiculopathy, a randomised controlled trial

**DOI:** 10.1186/1471-2474-15-129

**Published:** 2014-04-14

**Authors:** Sarita van Geest, Barbara Kuijper, Marinus Oterdoom, Wilbert van den Hout, Ronald Brand, Theo Stijnen, Pim Assendelft, Bart Koes, Wilco Jacobs, Wilco Peul, Carmen Vleggeert-Lankamp

**Affiliations:** 1 Department of Neurosurgery, Leiden University Medical Center (LUMC), P.O. Box 9600, NL-2300 RC Leiden, the Netherlands; 2 Department of Neurology, Maasstad Hospital Rotterdam, Rotterdam, the Netherlands; 3 Department of Neurosurgery, Martini Hospital, Groningen, the Netherlands; 4 Department of Medical Decision Making, LUMC, Leiden, the Netherlands; 5 Department of Medical Statistics, LUMC, Leiden, the Netherlands; 6 Department of Primary and Community care, Radboud University Hospital Nijmegen, Nijmegen, the Netherlands; 7 Department of General Practice, University Medical Centre Rotterdam, Rotterdam, the Netherlands

**Keywords:** Cervical herniated disc, Cervical radicular syndrome, Anterior discectomy, Foraminotomy, Cost-effectiveness, Conservative treatment, Randomized controlled trial, Shared decision making

## Abstract

**Background:**

Cervical radicular syndrome (CRS) due to a herniated disc can be safely treated by surgical decompression of the spinal root. In the vast majority of cases this relieves pain in the arm and restores function. However, conservative treatment also has a high chance on relieving symptoms. The objective of the present study is to evaluate the (cost-) effectiveness of surgery versus prolonged conservative care during one year of follow-up, and to evaluate the timing of surgery. Predisposing factors in favour of one of the two treatments will be evaluated.

**Methods/design:**

Patients with disabling radicular arm pain, suffering for at least 2 months, and an MRI-proven herniated cervical disc will be randomised to receive either surgery or prolonged conservative care with surgery if needed. The surgical intervention will be an anterior discectomy or a posterior foraminotomy that is carried out according to usual care. Surgery will take place within 2–4 weeks after randomisation. Conservative care starts immediately after randomisation. The primary outcome measure is the VAS for pain or tingling sensations in the arm one year after randomisation. In addition, timing of surgery will be studied by correlating the primary outcome to the duration of symptoms. Secondary outcome measures encompass quality of life, costs and perceived recovery. Predefined prognostic factors will be evaluated. The total follow-up period will cover two years. A sample size of 400 patients is needed. Statistical analysis will be performed using a linear mixed model which will be based on the ‘intention to treat’ principle. In addition, a new CRS questionnaire for patients will be developed, the Leiden Cervical Radicular Syndrome Functioning (LCRSF) scale.

**Discussion:**

The outcome will contribute to better decision making for the treatment of cervical radicular syndrome.

**Trial registration:**

NTR3504

## Background

The cervical radicular syndrome (CRS) caused by a cervical hernia nuclei pulposus (HNP), is a frequently occurring problem with an incidence of 0.8 per 1000 inhabitants per year and a prevalence of 3.5 per 1000 inhabitants [1,2]. CRS can lead to invalidating radicular pain and may lead to motor and sensory deficits [1,3]. In the majority of patients the symptoms gradually diminish within weeks to such an extent that normal daily activities can be continued [4-6].

Many controversies exist regarding the timing of diagnostic procedures, the timing of referral of patients to the neurologist or neurosurgeon and the timing of a surgical intervention for a cervical HNP [7]. Nowadays, the timing of an elective surgical intervention is not based on scientific knowledge, but on the preference of the individual neurologist and neurosurgeon and local production capacity.

A previous Dutch randomised controlled trial (RCT) compared different conservative strategies in patients with a short term CRS (at least 3 weeks pain in the arm). In all strategy arms the median visual analogue scale (VAS) pain in the arm decreased from 68–72 to 20–25 mm (range 0–50 mm) within six months [6]. Moreover, indirect evidence indicates that most cervical disc protrusions resolve spontaneously without surgery [6,8-11]. If complaints do not diminish spontaneously within reasonable time, surgery can be considered. Surgical intervention is usually performed by an anterior discectomy with subsequent anterior decompression of the spinal nerve. The effectiveness (pain relief, satisfaction, muscle weakness improvement) of the anterior discectomy to reduce symptoms is demonstrated to be between 80 and 95%, and the procedure has been established as a safe intervention [12-17]. Another approach to decompress the spinal nerve is a posterior foraminotomy which has a positive outcome in 75 to 95% of the cases [18]. Both conservative care and surgical intervention are successful treatments, but it is unknown which patients benefit most from each strategy [7-9,19]. Only two small RCTs have been performed; Persson *et al.* demonstrated that pain, function and mood after one year of follow-up was equal in the conservative and surgical group [20]. However, this was a small trial with 54 patients in the conservative and 27 patients in the surgery group and the power was estimated to be only 47%. Engquist *et al.* demonstrated that a higher percentage of patients that were operated (n = 31) rated their symptoms as ‘better’, than in the conservatively treated group (n = 32). This difference disappeared at the two year evaluation moment [21]. In both trials, timing of surgery was not studied, and no cost-effectiveness or predisposing factors were evaluated. Furthermore, two nonrandomised studies have been published; in one study (n(surgery) = 39; n(conservative) = 21) the outcomes were in favour of the conservative group after 5.5 years [22] while the other study (n (surgery) = 51; n (conservative) = 104) showed better results for the surgery group after 1 year follow-up [23]. However, the surgical group in both studies had more pain and worse functional scores at baseline, which excludes adequate outcome comparison.

No studies have been published regarding the cost-effectiveness of conservative and surgical therapy. In the Netherlands, on average 2000 patients yearly receive surgery for a cervical herniated disc, resulting in direct costs of about €30 million per year. Although direct costs for conservative care are lower, this group might have higher indirect costs due to a longer period of reduced labour productivity. Further research on cost-effectiveness could lead to reduction of costs for healthcare and society.

The primary goal of the present study is to compare the (cost-) effectiveness of conservative therapy for cervical HNP, sometimes followed by ‘late’ surgery if needed, with early surgical intervention. The timing of surgery will be studied, and several predisposing factors will be analysed. In addition to the trial, the participants will also participate in the development and validation of an outcome scale for CRS, the Leiden Cervical Radicular Syndrome Functioning (LCRSF) scale.

## Methods

### Design

The present study is a multi-centre patient-randomised controlled trial with parallel group design. The main research question will be answered after one year of follow-up and the complete follow-up will last two years. The Medical Ethics Committee of the Leiden University Medical Centre has approved the study protocol. Every participating centre independently has given approval before including patients. Informed consent will be obtained from all participating patients. The study was registered July 3, 2012 in the Dutch Trial register (NTR3504).

### Patients

All patients (18–75 yr.) with CRS and at least 2 months of disabling pain or tingling sensations will be eligible for participation. Either the VAS arm pain or the VAS tingling sensations should be more than or equal to 40 mm. MRI must confirm the presence of a herniated cervical disc as explanation for the symptoms. The inclusion and exclusion criteria are shown in Table 1.

**Table 1:** Inclusion and exclusion criteria

Inclusion criteria	age 18–75 years
	at least two months existing complaints of radicular sensations in the arm (VAS arm pain or VAS tingling sensations) of at least 40 mm
	herniated disc with root compression confirmed by MRI corresponding with the clinical symptoms
	signed informed consent
Exclusion criteria	paresis of MRC <4
	mainly signs of myelopathy
	any form of cervical spine surgery in the past
	instability of the cervical spinal column requiring stabilisation
	pregnancy
	insufficient knowledge of Dutch language
	planned emigration in the year after randomisation
	participation in another clinical trial

### Baseline data

The baseline questionnaires and demographics will be registered before randomisation. Also the preferences of patients and doctors will be registered using a 5-point scale varying from ‘strong preference for surgery’ to ‘strong preference for conservative treatment’.

### Randomisation procedure

A randomisation list is prepared for every participating hospital. Randomisation will take place in variable permutation blocks, which are unknown to the research nurse. A random generator is used to generate the random sequence within the blocks. The data manager, who is not involved in the selection and allocation of patients, will prepare coded and sealed envelopes containing the treatment allocation based on the pre-generated stratified random allocation list.

The research nurse will make an appointment with the participant after the visit to the neurologist or neurosurgeon. After registering baseline information, a sealed envelope with treatment allocation will be opened in front of the patient. Patients who do not wish to be randomised will be asked to participate in a cohort study, in which both surgically and conservatively treated patients will be followed.

### Interventions

#### Group A: Surgical intervention

Surgery will take place within 2–4 weeks of randomisation. ‘Usual care’ will be pursued. Therefore the surgeon can perform the surgery he prefers, with the exception of implantation of an artificial disc. Variables of the procedure are filled in a standardised form.

For anterior discectomy, the level of surgery is verified by fluoroscopy. The operation will be carried out by a qualified neurosurgeon. Most surgeons operate using loupe magnification. The platysma muscle is separated or cleaved at the right side of the midline (less frequently on the left side), and the prevertebral space is reached by an approach medial to the sternocleidomastoid muscle and the carotid artery, and lateral to the trachea and oesophagus. The disc is incided and the corpora are distracted. Discectomy is performed as thorough as possible. Regularly the posterior ligament is cut and the spinal root is decompressed. If necessary, spondylarthrotic rims are removed. To the preference of the surgeon bone graft or an intervertebral fusion device is left behind. Decompression of more levels is allowed. If the surgeon prefers to decompress the cervical nerve root from the dorsal side it is allowed to perform a dorsal foraminotomy. The muscles of the neck are unilaterally detached at the site of interest. With a high-speed drill a foraminotomy is performed to decompress the nerve.

The postoperative care will be performed according to ‘usual care’ and will regularly be a stay in the hospital for one or two days, without physiotherapeutic treatment. If possible most patients will gradually return to work within weeks after the surgical intervention.

#### Group B: Conservative management

Management of the conservative arm will be performed according to usual care by either the neurologist or general practitioner (GP). In the Netherlands, formal guidelines for this approach are lacking, however, related guidelines for lumbar radicular syndrome exist. The treatment of CRS is aimed primarily at pain relief and maintenance/restoration of normal day-to-day activities. Various studies have shown that adequate and unambiguous information about the problem from which the patient is suffering (the nature of the condition) and what the patient can expect (the prognosis), together with trustworthy counselling can reduce the anxiety and uncertainty felt by the patients Usually, no soft collar nor physiotherapy is prescribed, however it is allowed. A graded activity time schedule is made beforehand together with the patient [24,25].

In case of progressive neurological deficit or worsening intolerable pain the GP or neurologist can refer the patient back to the research nurse or neurosurgeon in order to consider surgery. If, six months after randomisation, the patient has still not improved or suffers from disabling intermittent CRS, surgical treatment will be offered.

### Outcome assessment

Non-blinded examinations by the research nurse will take place at baseline, at 6 weeks, 3, 6, 9, 12, and 24 months after randomisation by visits and filling in questionnaires (Table 2). In addition, the patients will keep a diary. A second MRI will be made one year after randomisation of which the outcome will be told after the assessments. Data will be recorded and analysed at the Data Coordination Centre in Leiden.

**Table 2:** **Follow**-**up flowchart**

	**Intake**	**Operation**	**6w**	**3 m**	**6 m**	**8 m**	**12 m**	**24 m**
Demography & diagnosis	X							
Neurological examination	X	X	x		x		x	x
Informed consent	X							
MRI	X						x	
NDI	X		x	x	x	x	x	x
VAS pain/tinglings	X		x	x	x	x	x	x
SF-36	X		x	x	x	x	x	x
IPQ-K	X							
Perceived recovery	X		x	x	x	x	x	x
Health state utility VAS	X		x	x	x	x	x	x
DS-14	X		x	x	x	x	x	x
EuroQol	X		x	x	x	x	x	x
Diary	X		x	x	x	x	x	x
Re-operation			x	x	x	x	x	x
Complication			x	x	x	x	x	x

Overview of follow-up moments and received information (for explanation: see text).

#### Primary outcome measure

The primary outcome parameter is severity of main complaint: VAS pain or tingling sensations. The disabling intensity in the arm will be measured with a 100 mm horizontal VAS; “no pain or tingling sensations” at 0 mm and “the most terrible pain or tingling sensations I can imagine” at 100 mm [26,27]. During each visit the VAS arm pain will be scored. Additionally, the time of complaints will be scored as the time between the first general practitioners visit for this pain in the arm till the moment of randomisation, measured in weeks.

#### Secondary outcome measures

1. Neck Disability Index (NDI). Although the NDI is a score to monitor complaints of cervical myelopathy, it gives a reliable impression of the disability of the patient with respect to daily activities [28,29]. The scores of the items are added up and transformed into a scale of 0 to 100. A higher score reflects a better health condition.

2. VAS pain or tingling sensations in the arm at different time points (intake, 6 weeks and 3, 6, 8, 24 months).

3. VAS pain in the neck. The pain intensity in the neck will be measured with a 100 mm horizontal VAS at different time points (intake, 6 weeks and 3, 6, 8, 12, 24 months).

4. Short Form-36 (SF-36). The SF-36 will be used as a generic quality-of-life questionnaire. The questionnaire relates to the analysis of the general functional status of patients. The scores of the different topics are added up and transformed into a scale of 0 to 100. A higher score reflects a better health condition. The different topics can be summarised in a physical and psychological main domain.

5. Illness Perceptions Questionnaire – short form (IPQ-K). The Brief Illness Perceptions Questionnaire is translated from English to Dutch and adapted for differences in culture [30]. This questionnaire represents the way a patient copes with his complaints, and it gives insight in the functioning of a patient.

6. Perceived recovery. To measure perceived recovery a seven-point Likert scale will be used [31]. The scores on this scale vary from ‘completely recovered’ to ‘worse than ever’. The scales will be completed by the patient, research nurse, and the neurologist or neurosurgeon.

7. Health state utility VAS. Patients will assess how they value their health using a VAS ranging from 0 = as bad as death to 100 = perfect health.

8. DS-14. This Dutch questionnaire determines the extent of ‘Distressed-personality’ of the patient. A type D personality is defined in medical psychology as the co-existent presence of both negative affectivity (e.g. worrying, irritability, gloom) and social inhibition (e.g. reticence and a lack of self-assurance). The questionnaire consists of seven questions concerning negative affectiveness and seven questions concerning social inhibition. Individuals that score on both dimensions 10 or higher are being classified as having a type D personality. A type D personality has been demonstrated to have a negative influence on outcome of disease [32,33].

9. EuroQol – EQ-5D. The EuroQol (EQ-5D) will be used to value the patients’ health for the cost utility analysis. As indicated by the name the tool measures five dimensions: mobility, self-care, daily activities, pain/discomfort, and anxiety/depression. Each domain is rated as having no problems, some problems or extreme problems.

10. Cost diaries, including absenteeism, will be filled out by the patients during follow-up.

11. MRI. MRI findings at baseline and after 1 year will be compared: extrusion or protrusion, the difference in size of the disc herniation, nerve root compression, and, if operated, presence of scar tissue will be registered. The data will be registered using standardised CRFs, which will be filled out by the local radiologist and neurosurgeon.

12. Frequency of (re)operation in the conservative group and frequency of reoperation in the surgery group.

13. Frequency of complications.

### Sample size

For the primary research question, the sample size is based on the standard deviation of VAS arm pain, which is estimated between 25 and 30, as reported by Persson after 1 year of follow-up [20]. If we consider an α = 0.05 and a power of 90%, and we take 15 mm difference in VAS pain in the arm as clinically relevant, then we need 100 patients in both groups (including a 10% compensation for lost to follow-up) in a worst case scenario of a standard deviation of 30 to show such a difference as statistically significant under the null hypothesis. Because the analysis for the secondary question requires more patients than the primary research question, the sample size is chosen with regard to the secondary research question. In order to investigate the modifying effect of the (pre-randomisation) duration of pain (X, in months) on the treatment effect (T) a covariance-analysis will be performed with the VAS measured after 12 months follow-up as a dependent variable and T, X and the interaction T*X as independent variables. The coefficient of T*X represents the increase (or decrease) of the treatment effect per month duration of pain on baseline. The standard error of the slope of this regression line is $\sqrt{\left(1-\rho^{2}\right)}\sigma_{\mathrm{vas}}/\left(\sqrt{n-1}\sigma_{x}\right)$, in which ρ is the correlation between the duration of pain at baseline and the VAS after 12 months follow-up, σ_VAS_ the SD of the VAS after 12 months follow-up and σ_X_ the SD of the duration of pain at baseline. Based on the RCT by Persson [20] the σ_VAS_ is estimated conservatively at 30 mm and the SD of the duration of pain at baseline conservatively at 25. We are not informed about the correlation, but ρ = 0 leads to a conservative estimate of the standard error of the slope. Because this correlation is probably small, the overestimation of the sample size is probably limited (for instance 1% if ρ = 0.1 or 4% if ρ = 0.20). The number of patients needed for a power 1-β = 0.80 and two-sided significance level of α = 0.05 can be calculated as $n_{\mathrm{pergroup}}-1={\left(1.96+0.84\right)}^{2}\cdot 2\sigma^{2}/\Delta^{2}$, in which σ = σ_VAS_/σ_X_ and Δ is the increase of the difference in slope per month. An increase in the difference in slope of 4 mm per year duration of pain at baseline is considered as clinically relevant. According to the formula above, 200 patients per study arm are needed, adding up to a total of 400 patients.

The number of 200 patients in both groups that is needed for the second primary research question also has the advantage that it adds some additional power for subgroup analyses. By taking 200 patients per group the power for the first research question rises to above 99% for a SD of 30 and a difference of 15.

### Statistical analysis

All base case analyses will be performed based on the ‘intention to treat’ principle. Analysis will however, also be performed based on a ‘as treated’ analysis.

#### Primary outcome

A linear mixed model will be used for the primary outcome measure VAS pain/ tingling sensations in the arm. Covariates in the model will be time as categorical, treatment and the interaction between time and treatment. The covariance model will be unstructured. The main endpoint parameter, the difference between the two arms at 1 year, will be estimated from this model. To answer the question whether the difference in effect between surgical and conservative treatment depends on de duration of complaints, this linear mixed model will be extended with extra (interaction) terms.

#### Secondary outcome

Secondary outcomes will be analysed for differences between groups with linear mixed models similar to the main endpoint. Furthermore, we will investigate whether potential prognostic variables (gender, age, presence of neck pain, more neck pain than arm pain, Valsalva manoeuvre test, Spurling test, shoulder abduction test, type of personality based on DS-14 and based on IPQ-K, anterior or posterior approach, smoking, neurological deficits (strength on an 1–5 Medical Research Council (MRC) scale and sensibility), duration of the symptoms, Quetelet index) are associated with the difference between the treatment arms in the primary endpoint. This will be carried out by adding a potential prognostic variable and its interaction with the treatment arm to the linear mixed model.

#### Cost-effectiveness

The economic evaluation will be a cost-utility analysis from a societal perspective, based on patient reports and with a 1-year time horizon (without discounting). The analysis will be very similar to the analysis performed for the lumbosacral radicular syndrome [34].

Group averages will be compared using double-sided bootstrapping and costs will be compared to patient outcome using net-benefit analysis. An integral cost–price analysis will be performed to estimate operating costs. Other health care costs will be valued using standard prices [35], including time and travel costs. Absenteeism will be valued using both the friction-cost method (primary analysis) and the human-capital method. Quality-adjusted life years (QALYs) will be estimated using the EQ5D (primary analysis, Dutch tariff), the SF6D and the patients’ health state utility VAS (transformed to a utility scale using the power transformation U = 1-(1-VAS/100)^1.61^) [36].

### Development CRS questionnaire

The participants will also participate in the development and validation of a functioning scale for CRS: the Leiden Cervical Radicular Syndrome Functioning (LCRSF) scale. This questionnaire will combine arm pain, neck pain, and motor and sensory deficits. A secondary aim is to build a prediction model predicting SF-6D and EQ-5D utilities from the LCRSF scale.

### Timeline

Ethics approval was obtained in May 2012 from the Human Research Ethics Committee of the Leiden University Medical Center. Approval from the participating hospitals occurred during May 2012 – now. Recruitment of participants commenced in August 2012. All participants are expected to have completed the study by the summer of 2016.

## Conclusion and Discussion

The objective of the current trial is to study the effectiveness of the surgical intervention and prolonged conservative care for CRS. Secondary, an analysis of cost-effectiveness, timing of surgery and prognostic factors in favour of one of the strategy arms will be performed. The only two other randomised trials on this subject enrolled a limited number of patients and did not measure these secondary outcomes.

The CASINO trial is performed using ‘usual care’ and has a pragmatic design. It acknowledges that sometimes it may not be possible to postpone surgery for every conservative care patient until 6 months after allocation and that some patients will recover before surgery is performed in the surgical group. In these cases we consider it infeasible and unethical to hold on to the randomised treatment. Because of the intent-to-treat analysis, these cases will be analysed according to the originally allocated randomisation arm. Methodologically, this is the appropriate way to compare two healthcare strategies, as opposed to two treatments.

We hypothesise that prolonged conservative care will have comparable results as surgery on primary outcome (i.e. VAS pain or tingling sensations) after one year of follow-up. However, due to the risks and the costs of surgery we hypothesise that prolonged conservative care will be more (cost-) effective with less adverse events.

We hypothesise that those patients who endured symptoms for a longer time, would benefit from surgery. If true, it would be interesting to investigate whether there is a cut-off time-point after which surgery demonstrates a better result.

Finally, the predefined subgroup analyses may provide us with parameters, which can predict preference for one of the two treatment strategies. It is hypothesised that patients demonstrating more neck pain than arm pain or a type D personality will have better outcomes with conservative therapy.

The results of the present study will provide insight in the results of surgery at an early phase and of prolonged conservative care. This information will be useful in shared decision-making for choosing the most appropriate treatment for radicular cervical syndrome.

## Abbreviations

CRS: Cervical radicular syndrome; DS-14: Distressed personality questionnaire; GP: General practitioner; HNP: Hernia nuclei pulposus; IPQ-K: Illness perception questionnaire; MRC: Medical research council; MRI: Magnetic resonance imaging; NDI: Neck disability index; QALY: Quality-adjusted life year; RCT: Randomised controlled trial; SF-36: Short form-36; VAS: Visual analogue scale.

## Competing interest

The authors declare that they have no competing interests with respect to the current trial.

## Authors’ contributions

SG is the principal investigator of the trial. BKu and MO are local investigators that participate in the review process of this article, WH is co-designer of the protocol and responsible for the cost-effectiveness study, RB and TS are the biostatisticians involved with the trial and are also responsible for the implementation of the trial data management using ProMISe software, WA and BKo are co-designers of the protocol and deliver input from a general practitioners’ point of view. WJ is responsible for the epidemiological design of the trial, WP is co-designer of the protocol and co-responsible for the RCT. CV is the designer of the protocol and has final responsibility for the RCT. All authors participated in the trial design and coordination. All authors read and approved the final manuscript.

## Pre-publication history

The pre-publication history for this paper can be accessed here:

http://www.biomedcentral.com/1471-2474/15/129/prepub
